# Data on the relationship between internet addiction and stress among Lebanese medical students in Lebanon

**DOI:** 10.1016/j.dib.2019.104198

**Published:** 2019-08-06

**Authors:** A. Samaha, M. Fawaz, A. Eid, M. Gebbawi, N. Yahfoufi

**Affiliations:** a Lebanese University, Doctoral School for Literature and Social Sciences, Lebanon; b Beirut Arab University, Faculty of Health Sciences, Lebanon; c Department of Biomedical Sciences, Lebanese International University, Lebanon; d Lebanese University, Faculty of Public Health, Lebanon; e Department of Pharmacology and Toxicology, Faculty of Medicine, American University of Beirut, Lebanon; f Department of Pediatrics and Adolescent Medicine, American University of Beirut, Lebanon

**Keywords:** Internet addiction, Stress, Medical students, Lebanon

## Abstract

Stress and behavioral addiction are becoming major health problems growing in strength and prevalence. They are often associated with a large array of debilitating diseases and conditions including psychosocial impairments. Medical students remain a vulnerable territory for developing stress and addiction mainly relating to Internet use. Data was gathered from medical students around Lebanon on the relationship between stress and internet addiction. The data in this article provides demographic data about medical students in Lebanon, their stress levels, sources of stress as well as the level of internet addiction recorded in relation to their stress levels. The analyzed data is provided in the tables included in this article.

**Table:** Specifications Table

Subject area	*Psychology*
More specific subject area	*Stress and Addiction*
Type of data	*Tables*
How data was acquired	*Quantitative – Surveys: MSSQ and IAT Questionnaires*
Data format	*Raw and Analyzed*
Experimental factors	A correlational cross-sectional design was adopted.
Experimental features	An array of standardized questionnaires were used to collect quantitative data. The questionnaires were the Internet Addiction Test (IAT), Medical Student Stressor Questionnaire (MSSQ), and Caffeine consumption and dependence Scale.
Data source location	*Data was collected from various medical schools in Beirut, Lebanon*
Data accessibility	*Summary statistics available within this article*
Related research article	*Chaudhari B, Menon P, Saldanha D, Tewari A, Bhattacharya L. Internet addiction and its determinants among medical students. Industrial psychiatry journal. 2015 Jul;24(2):158.*[1]

**Table:** 

**Value of the data** • The data provided is of value as no large studies have been conducted to determine the prevalence of stress in the Lebanese medical students and its impact on addictive behaviors and performance. • Other researchers conducting similar studies might find this study very useful in order to have a reference for comparison of results, as well as a base to conduct further studies which would explore this phenomena among medical students. • The data might also be used as a base for experimental studies as well, where researchers can use the produced results to design intricate experiments that could be addressing this issue.

## 1 Data

The mean age of the participants is 21.92 ± 2.16 years. The majority of respondents belong to the first four academic years (462 students) (Table 1). Among all participants 126 (21.15%) students have one or both parents working as physicians and 78 (13.08%) have one or both parents working in a health-related fields (Table 2). The majority of students belong to families with an average income between 1000 and 3000 US dollars (43.95%), with 35.92% of families earning a monthly income of more than 3000 USD and 20.13% of families earn less than 1000 US dollar per month as shown in Table 3.

**Table 1:** Recruited students per academic year.

Level of studies	Number of participants	Percentage
1st year	152	25.5
2nd year	96	16.1
3rd year	104	17.44
4th year	110	18.45
5th year	60	10.06
6th year	74	12.45
Total	596	100

**Table 2:** Parents’ work and education.

Parents' work and education level	Mothers		Fathers	
	N	%	N	%
Working	248	41.61	546	91.61
Housewife/Unemployed	348	58.39	50	8.38
Illiterate	16	2.68	22	3.69
Primary education	82	13.75	88	14.76
Secondary education	164	27.51	122	20.46
University level	178	29.86	168	28.18
Higher education	156	26.17	196	32.88

**Table 3:** Average monthly income by all family members in US dollars.

Average family monthly income	N	%
Less than 1000	120	20.13
Between 1000 and 3000	262	43.95
Between 3000 and 6000	142	23.84
More than 6000	72	12.08

The majority of participants have their tuition fees managed by their families (87.91%) with partial contribution from students themselves in rare occasions (2.35%) or from scholarships (7.72%) as noted in Table 4.

**Table 4:** Management of study fees.

Who manage study tuition	N	%
Family	524	87.91
Students partial contribution	14	2.35
scholarship	46	7.72
Others	12	2.02

**Table 5:** MSSQ perceived stressors.

Level of stress	ARS		IRS		TLRS		SRS		DRS		GRAS	
	N	%	N	%	N	%	N	%	N	%	N	%
None	24	4	24	4	22	3.7	28	4.7	14	2.3	26	4.4
Mild	46	7.7	232	39.3	204	34.2	158	26.5	368	61.7	188	31.5
Moderate	130	21.8	172	28.9	184	30.9	224	37.6	124	20.8	202	33.9
High	206	34.6	124	20.8	142	23.8	128	21.5	48	8.1	134	22.5
severe	190	31.9	44	7	44	7.4	58	9.7	42	7	46	7.7
Total	596		596		596		596		596		596	

The stress domains among medical students have been measured using the Medical Student Stressor Questionnaire [2]. Additional information about the MSSQ is found in the Questionnaire section. The Data of the MSSQ revealed that 396 students (66.44%) are subject to high and severe Academic Related Stress (ARS) and that 31.2% report equally high and severe stress related to Teaching and Learning Related Stress (TLRS) and Social Related Stressors (SRS) domains. Group Related Activity (GRAS) is reported high and severely stressful by 180 students (30.2%). The Intrapersonal Related Stressor (IRS) and the Drive and Desire Related Stressor (DRS) seem to have minor effects on students; respectively 168 (28.18%) and 90 (15.1%) students report them as causing high and severe stress.

Taking into consideration the general average of each stress domain as calculated using Medical Student Stressor Questionnaire (MSSQ) [2], few significant results were noted (see Table 5). An Independent T test was carried out and that data shows that there is a significant difference related to gender between ARS, IRS and TLRS where females have endorsed higher levels of stress in the three domains where the recorded means were 2.8 for ARS, 1.67 for IRS and 1.85 for TLRS. An ANOVA test was carried out and the data shows that a significant difference is noted between income groups and ARS and IRS; in both domains students belonging to high-income families are less subjected to stress in both domains. These results are reflected in Table 6, Table 7 respectively.

**Table 6:** Stress domains and gender.

Stress domain	Gender	Mean ± Standard deviation	P value
ARS	Male	2.234 ± 1.038	<0.00
	Female	2.815 ± 0.838	
IRS	Male	1.233 ± 1.041	0.00
	Female	1.619 ± 1.103	
TLRS	Male	1.441 ± 1.033	0.00
	Female	1.825 ± 1.028	
SRS	Male	1.572 ± 0.959	0.19
	Female	1.876 ± 1.032	
DRS	Male	0.856 ± 1.052	0.26
	Female	1.571 ± 1.005	
GARS	Male	1.571 ± 1.005	0.8
	Female	1.808 ± 0.961

**Table 7:** Stress domains and income.

Stress domain	Income in USD	Mean ± Standard deviation	P value
ARS	<1000	2.390 ± 0.927	0.00
	1000–3000	2.617 ± 0.899	
	3000–6000	2.933 ± 0.834	
	>6000	2.161 ± 1.200	
IRS	<1000	1.571 ± 1.052	0.00
	1000–3000	1.696 ± 1.059	
	3000–6000	1.148 ± 1.079	
	>6000	1.021 ± 1.050	
TLRS	<1000	1.503 ± 1.162	0.18
	1000–3000	1.748 ± 0.98	
	3000–6000	1.772 ± 1.029	
	>6000	1.361 ± 1.171	
SRS	<1000	1.659 ± 0.849	0.56
	1000–3000	1.828 ± 1.061	
	3000–6000	1.902 ± 1.058	
	>6000	1.313 ± 0.895	
DRS	<1000	0.837 ± 1.056	0.14
	1000–3000	1.246 ± 1.168	
	3000–6000	0.927 ± 1.128	
	>6000	1 ± 1.156	
GARS	<1000	1.479 ± 0.897	0.06
	1000–3000	1.784 ± 0.964	
	3000–6000	1.919 ± 0.985	
	>6000	1.259 ± 0.982	

More than half of the participants (57.39%) spend more than 3 h online on a daily basis as noted in Table 8.

**Table 8:** Daily time spent online.

Daily time	N	%
Less than half hour	46	7.72
0.5–1 hour	52	8.72
1–3 hours	156	26.17
3–5 hours	162	27.18
More than 5 hours	180	30.21

The impact of internet use on participants’ life and its addiction pattern are evaluated using Internet Addiction Test (IAT) scale. 156 participants (26.1%) belong to risky group as internet use may negatively affect their life and precipitate for significant problems. Table 9 shows the distribution of internet users among the various levels of addiction scored.

**Table 9:** Internet addiction test IAT.

Level of addiction	N	%
None (IAT score 0–30)	150	25.2
Average online user (IAT score 31–49)	290	48.7
Frequent problems and negative impact on life (IAT score 50–79)	148	24.8
Significant life problems (IAT score 80–100)	8	1.3

A Pearson's correlation test was conducted and the data shoes that the Internet Addiction Score is not significantly related to the collected participants' demographics as mentioned in Table 10. Therefore, analysis will be based on analyzing correlation between IAT and different study variables. There is no significant difference between males and females concerning internet use. Likewise, financial issues and parents' statuses are found to have no significant effects on internet addiction. The correlation is very weak and non-significant between IAT and different stress domains.

**Table 10:** IAT and demographics.

Demographics		IAT Mean ± standard deviation	P value
Gender	Male	39.577 ± 18.352	0.76
	Female	40.506 ± 17.603	
Income in US dollars	<1000	35.428 ± 16.159	0.63
	1000–3000	40.787 ± 18.622	
	3000–6000	43.603 ± 18.073	
	>6000	34.545 ± 16.029	
Parents' status	Both alive	39.932 ± 18.097	0.647
	One alive	43.642 ± 12.067	
	None alive	55 ± 0.01	
	Divorced	34.5 ± 21.977	

A multiple linear regression model was constructed to see how Internet addiction (IAT) as quantitative dependent variable varies according to stress domains, controlling for possible confounding variables (gender, smoking status, and income. Dummy variables were created for gender, smoking status, and categories of income. However, before running Multivariate Regression Analysis, the following assumptions were tested:

1. The first assumption that was tested is that the dependent variable was measured on a continuous scale. Given that the dependent variable in this case is the Internet Addiction Score, this assumption was met.
2. The second assumption to be tested was that to run a multiple linear regression, two or more independent variables should be included and this assumption was easily met.
3. The third assumption that was to run a multiple linear regression model, each independent variable has to have at least 20 records or entries, and considering the sample size of our study (N = 256), this assumption seems to be clearly met.
4. The fourth assumption was that the model should have independent observations or independence of residuals. This was proven by the Durbin-Watson test where an R-squared of 0.15 was noted therefore meeting the assumption at hand.
5. The fifth assumption to be tested was normality or normal distribution of the outcome variable; in this case Internet Addiction Score. For that matter a Shapiro-Wick test was carried out. The results of this test showed that there is a statistically non-significant figure P = 0.49 which is greater than 0.05, therefore the dependent variable in this model is normally distributed thus meeting the assumption. The Kolmogorov-Smirnov test also showed a non-significant P-value of 0.20 which also proves the normal distribution of the dependent variable (Table 11).

**Table 11:** Test of normality.

	Kolmogorov-Smirnova			Shapiro-Wilk		
	Statistic	df	Sig.	Statistic	df	Sig.
IATtotal	.042	256	.200*	.995	256	.494
6. The sixth assumption to be tested was the absence of multicollinearity among the independent variables. This was done through checking the correlation coefficients and Tolerance/VIF values. The results showed that all correlation coefficients among the independent variables was below 0.7, therefore proving that the independent variables are not multicollinear and thus passing the mentioned assumption.
7. The seventh assumption to be tested was that the independent variables have a linear relationship with the dependent variable, attaining homoscedasticity. This is tested through checking the Normal Probability-Probability Plot, where it shows that the placements of the independent variables are falling across the line of the dependent variable.

**Figure:**
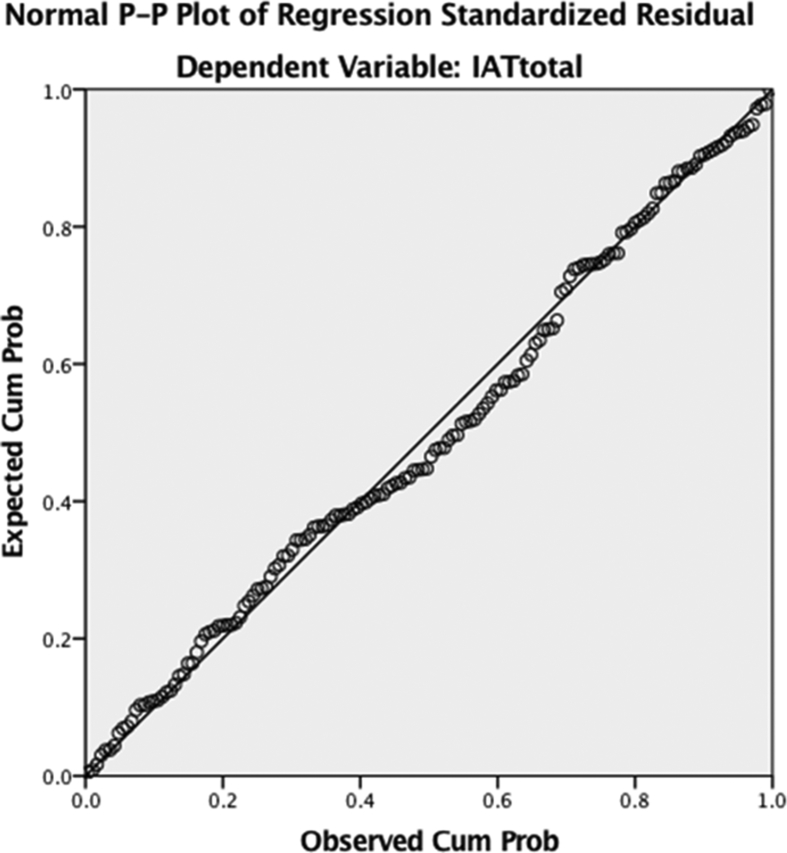

8. The eighth assumption to be tested is that there is no significant outliers, high leverage points, or high influential points. Upon analysis, the scatterplot shows that all the points lei between 3 and -3, therefore meeting this assumption.

**Figure:**
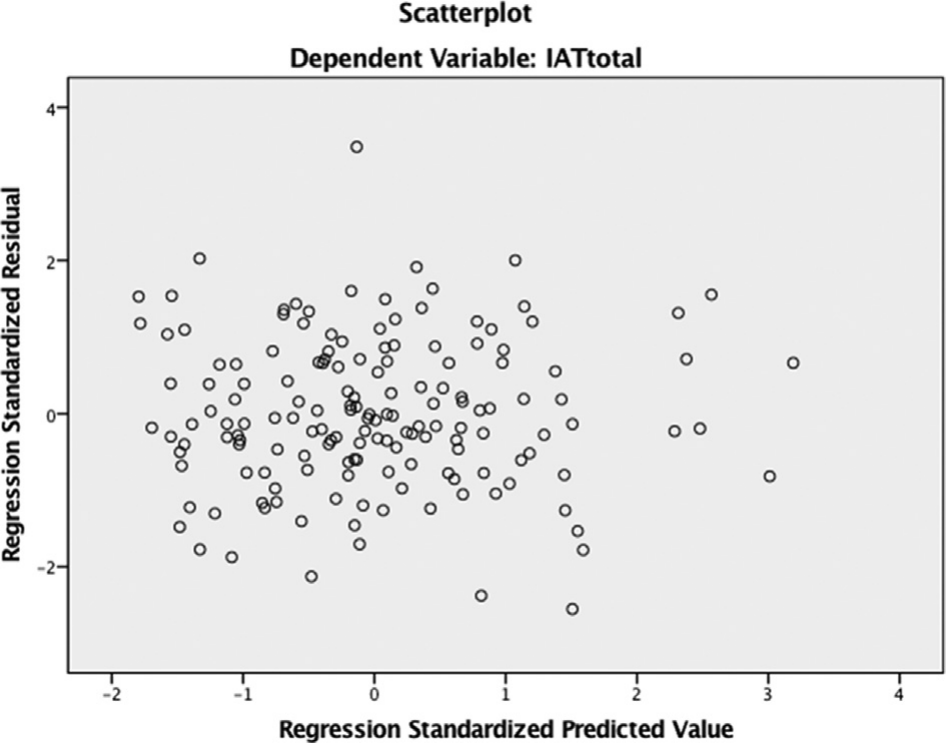

9. The final assumption to be tested is that the residuals are approximately normally distributed. The results show that the minimum residual is −2.5 and the maximum standard residual is −3, Cook's distance also recorded a minimum of 0.00 and a maximum of 1.08. Therefore the residuals are approximately normally distributed.

After meeting all the assumptions, the multiple regression model with Internet Addiction Score as a dependent variable is analyzed. The reference groups were selected as follows for each of the dummy variables respectively: male, no smoker, income higher than 1000 US dollars. The groups included in the model were compared to the reference groups.

The data shows that DRS maintained significant relation with IAT (p = 0.045) and the group with income less than 1000 USD appeared to have higher risk to have higher IAT scores (beta = −0.36, p = 0.03) (See Table 12).

**Table 12:** IAT Regression model.

Model	Standardized Coefficients	Sig.	95% Confidence Interval for B	
	Beta		Lower Bound	Upper Bound
(Constant)		.147	−16.409	103.936
ARS	.209	.352	−5.665	15.393
IRS	−.380	.103	−19.292	1.885
TLRS	−.116	.622	−13.096	7.971
SRS	.186	.364	−5.271	13.903
DRS	.396	.045	−.694	14.879
GARS	.147	.530	−6.730	12.783
Female	.093	.562	−10.307	18.572
Smoker	.117	.519	−14.710	28.460
Income < 1000 USD	−.359	.035	−43.223	−1.727

## 2 Experimental design, materials, and methods

### 2.1 Design

A quantitative approach using descriptive correlational cross-sectional design was adopted.

### 2.2 Sample and settings

A convenience sample of medical students enrolled in different studying years in different Lebanese universities is adopted in this study. A total of 800 students were approached to participate in this study, 720 of them consented for enrollment (90% respond rate) and 596 students have completed appropriately and fully the questionnaire to be suitable for analysis. The students were approached by the researcher, where the aim pf the study was explained and they were asked to sign an informed consent, and then fill the paper-based questionnaires after explaining the items. The students were sampled from medical schools that follow the Lebanese educational system, where students need to finish 6 years of education to graduate as general physicians.

### 2.3 Questionnaires

#### 2.3.1 Medical Student Stressor Questionnaire (MSSQ)

The short version of MSSQ used in the current study consists of 20 items representing the six main stressor domains studied among medical students. A validation study was conducted on 761 medical students representing multiple ethnicities, religions and cultures [3]. The validation established that the MSSQ has good psychometric properties; it is a valid and consistent instrument that can be used to identify students’ stressors as well as measure the intensity of the stressors. Reliability analysis shows that the MSSQ has a high internal consistency [4]. Stressors are grouped in six hypothetical groups: academic related stressors (ARS), intrapersonal and interpersonal related stressors (IRS), teaching and learning-related stressors (TLRS), social related stressors (SRS), drive and desire related stressors (DRS), and group activities related stressors (GARS). Based on score analysis perceived stress in each category is classified as mild, moderate, high and severe with respective scores of 0.00–1.00, 1.01–2.00, 2.01–3.00 and 3.01–4.00.

#### 2.3.2 Internet Addiction Test (IAT)

The Internet Addiction Test (IAT) was created by Young (1998) to evaluate the existence and intensity of Internet addiction, in a North American population sample [5]. The tool encompasses various Internet use demeanors and recurrent addiction indicators, with the noteworthy exclusion of tolerance. The instrument comprises 20 items; each was extracted from previous studies and clinical research on obsessive online consumers and their features. These 20 elements evaluate attributes and demeanors related to obsessive consumption of the Internet that comprises compulsivity, escapism, and dependency; noting that being online is any act that includes using the internet or network. Questions are randomized and each statement is weighted along a Likert-scale continuum that ranges from 0 = less extreme behavior to 5 = most extreme behavior for each item.

### 2.4 Statistical analysis

For the quantitative component collected data entry and analysis were performed using Statistical Package for the Social Sciences [6]. Descriptive results are reported as means and standard deviations or as percentages. Correlational Bi- and Multi-variate analyses were used to assess relationships between studied variables. Significance of results is defined according to Pearsons’ coefficient of correlation and P value. The threshold for significance was set at 0.05.
