# Supplementary material for: Data on the relationship between internet addiction and stress among Lebanese medical students in Lebanon
Source: Data Brief. 2019 Aug 6;25:104198. doi: 10.1016/j.dib.2019.104198 (PMC6706676; doi:10.1016/j.dib.2019.104198)
Supplement: Supplementary file 2 [file mmc2.pdf]

Internet Addiction Test (IAT)

From netaddiction.com

The Internet Addiction Test (IAT) is the first Validated measure of Internet Addiction described in the **IAT Manual** to measure Internet use in terms of mild, moderate, to several levels of addiction. For more information on using the IAT and building an Internet Addiction treatment program in your practice, visit **RestoreRecovery.net** for our comprehensive workbook and training programs. Based upon the following five-point likert scale, select the response that best represents the frequency of the behavior described in the following 20-item questionnaire.

0 = Not Applicable

1 = Rarely

2 = Occasionally

3 = Frequently

4 = Often

5 = Always

1. ___How often do you find that you stay online longer than you intended?
2. ___How often do you neglect household chores to spend more time online?
3. ___How often do you prefer the excitement of the Internet to intimacy with your partner?
4. ___How often do you form new relationships with fellow online users?
5. ___How often do others in your life complain to you about the amount of time you spend online?
6. ___How often do your grades or school work suffer because of the amount of time you spend online?
7. ___How often do you check your e-mail before something else that you need to do?
8. ___How often does your job performance or productivity suffer because of the Internet?
9. ___How often do you become defensive or secretive when anyone asks you what you do online?
10. ___How often do you block out disturbing thoughts about your life with soothing thoughts of the Internet?
11. ___How often do you find yourself anticipating when you will go online again?
12. ___How often do you fear that life without the Internet would be boring, empty, and joyless?
13. ___How often do you snap, yell, or act annoyed if someone bothers you while you are online?
14. ___How often do you lose sleep due to late-night log-ins?
15. ___How often do you feel preoccupied with the Internet when off-line, or fantasize about being online?
16. ___How often do you find yourself saying “just a few more minutes” when online?
17. ___How often do you try to cut down the amount of time you spend online and fail?
18. ___How often do you try to hide how long you’ve been online?
19. ___How often do you choose to spend more time online over going out with others?
20. ___How often do you feel depressed, moody, or nervous when you are off-line, which goes away once you are back online?

After all the questions have been answered, add the numbers for each response to obtain a final score. The higher the score, the greater the level of addiction and creation of problems resultant from such Internet usage. The severity impairment index is as follows:

NONE 0 – 30 points

MILD 31- 49 points: You are an average online user. You may surf the Web a bit too long at times, but you have control over your usage.

MODERATE 50 -79 points: You are experiencing occasional or frequent problems because of the Internet. You should consider their full impact on your life.

SEVERE 80 – 100 points: Your Internet usage is causing significant problems in your life. You should evaluate the impact of the Internet on your life and address the problems directly caused by your Internet usage.
